# A synthetic-lethality RNAi screen reveals an ERK-mTOR co-targeting pro-apoptotic switch in *PIK3CA*^+^ oral cancers

**DOI:** 10.18632/oncotarget.7372

**Published:** 2016-02-13

**Authors:** Kosuke Yamaguchi, Ramiro Iglesias-Bartolomé, Zhiyong Wang, Juan Luis Callejas-Valera, Panomwat Amornphimoltham, Alfredo A. Molinolo, Ezra E. Cohen, Joseph A. Califano, Scott M. Lippman, Ji Luo, J. Silvio Gutkind

**Affiliations:** ^1^ Moores Cancer Center, University of California San Diego, San Diego, CA, USA; ^2^ Laboratory of Skin Biology, National Institute of Arthritis and Musculoskeletal and Skin Diseases, National Institutes of Health, Bethesda, MD, USA; ^3^ Laboratory of Cancer Biology and Genetics, Center for Cancer Research, National Cancer Institute (CCR-NCI), National Institutes of Health, Bethesda, MD, USA; ^4^ Oral and Pharyngeal Cancer Branch, National Institute of Dental and Craniofacial Research, National Institutes of Health, Bethesda, MD, USA

**Keywords:** synthetic lethality screen, shRNA library, rapamycin, trametinib, co-targeting therapy

## Abstract

mTOR inhibition has emerged as a promising strategy for head and neck squamous cell carcinomas (HNSCC) treatment. However, most targeted therapies ultimately develop resistance due to the activation of adaptive survival signaling mechanisms limiting the activity of targeted agents. Thus, co-targeting key adaptive mechanisms may enable more effective cancer cell killing. Here, we performed a synthetic lethality screen using shRNA libraries to identify druggable candidates for combinatorial signal inhibition. We found that the ERK pathway was the most highly represented. Combination of rapamycin with trametinib, a MEK1/2 inhibitor, demonstrated strong synergism in HNSCC-derived cells *in vitro* and *in vivo*, including HNSCC cells expressing the *HRAS* and *PIK3CA* oncogenes. Interestingly, cleaved caspase-3 was potently induced by the combination therapy in *PIK3CA^+^* cells *in vitro* and tumor xenografts. Moreover, ectopic expression of *PIK3CA* mutations into *PIK3CA^−^* HNSCC cells sensitized them to the pro-apoptotic activity of the combination therapy. These findings indicate that co-targeting the mTOR/ERK pathways may provide a suitable precision strategy for HNSCC treatment. Moreover, *PIK3CA*^+^ HNSCC are particularly prone to undergo apoptosis after mTOR and ERK inhibition, thereby providing a potential biomarker of predictive value for the selection of patients that may benefit from this combination therapy.

## INTRODUCTION

Recent advances in RNAi technology have enabled synthetic lethal screenings in mammalian cells on a genome-wide scale [1]. Synthetic lethality, first described in yeast genetics, occurs when alteration of a gene results in change of the cellular phenotype only in the presence of another nonlethal genetic alteration. Recently, this approach has been applied to mammalian cells using RNAi screens [2, 3]. For example, RNAi screens in combination with active compounds were used for the identification of sensitizing targets and novel genetic interdependencies in cancer [4, 5]. RNAi screens can be carried out with either siRNA-based transient transfection or shRNA-based stable gene knockdown. Vector-based shRNA libraries have several unique advantages that make them particularly attractive: they can be screened in pools and this significantly reduces the cost of the screen; they afford long-term gene knockdown and thus can reveal slow phenotypic changes in the cell; and they can be readily adapted for *in vivo* screens in mouse models [1].

Head and neck squamous cell carcinomas (HNSCC) are among the ten cancers most frequently diagnosed each year in the United States, affecting approximately 42,000 new patients and resulting in approximately 8,300 deaths [6]. Mammalian target of rapamycin (mTOR) is at the center of signaling pathways that are critical for the regulation of cellular metabolism, growth, and proliferation [7]. Recent findings indicate that multiple genetic and epigenetic alterations converge on the persistent activation of PI3K/AKT/mTOR signaling in most HNSCC lesions [8-13]. Specifically, activating mutations in the PI3K catalytic subunit α, encoded by the *PIK3CA* gene, is the most frequent oncogenic event in HNSCC, with 18.1 % of all HNSCC displaying *PIK3CA* mutations and 21.2 % of cases displaying *PIK3CA* gene copy number gain. In addition, HNSCC also have multiple genomic alterations, such as *PTEN* mutations (2.8 %) and gene copy number loss (31.0 %) and activating mutations in *RAS* (5.9 %) and *AKT* (2.2 %) genes that result in PI3K/mTOR pathway activation. This cancer driver overreliance may in turn render HNSCC particularly sensitive to PI3K and mTOR inhibitors. Indeed, we and others have demonstrated this pathway dependence in a large series of genetically-defined and chemically-induced preclinical HNSCC experimental models by inhibiting mTOR with rapamycin and its analogs, which inhibit the activity of mTORC1 *via* binding to FKBP-12 and forming a ternary com­plex with mTOR [14-18].

The use of rapamycin and rapalogs have validated the concept that the PI3K/AKT/mTOR pathway can be successfully targeted in clinical cancer treatment. In this regard, our recently completed clinical trial using rapamycin in newly diagnosed and previously untreated HNSCC patients has demonstrated promising clinical activity [19] in contrast to most tumor types in which rapalogs often have modest and highly variable responses [18]. However, most targeted agents promote the activation of adaptive cellular responses that ultimately render cancer lesions resistant to their antitumor effect [20]. Thus, the combinatorial use of mTOR inhibitors with other drugs interfering with these resistance mechanisms may represent a promising strategy to improve treatment efficacy. In order to identify new potential targets for combination treatment with mTOR inhibitors, we performed a synthetic lethality screen using a pooled shRNA library with rapamycin in HNSCC cells. We identified a synthetic lethal interaction between ERK pathway inhibition and rapamycin, and validated the synergism of the co-target treatment on the growth inhibition of HNSCC cells *in vitro* and *in vivo*. Furthermore, we found that HNSCC cells harboring *PIK3CA* mutations are particularly susceptible to undergo apoptosis upon mTOR and ERK inhibition, thus providing a new therapeutic option for *PIK3CA*^+^ HNSCC patients.

## RESULTS

### An RNAi screens reveals synthetic lethal interactions with mTOR inhibitors

**Figure 1:**
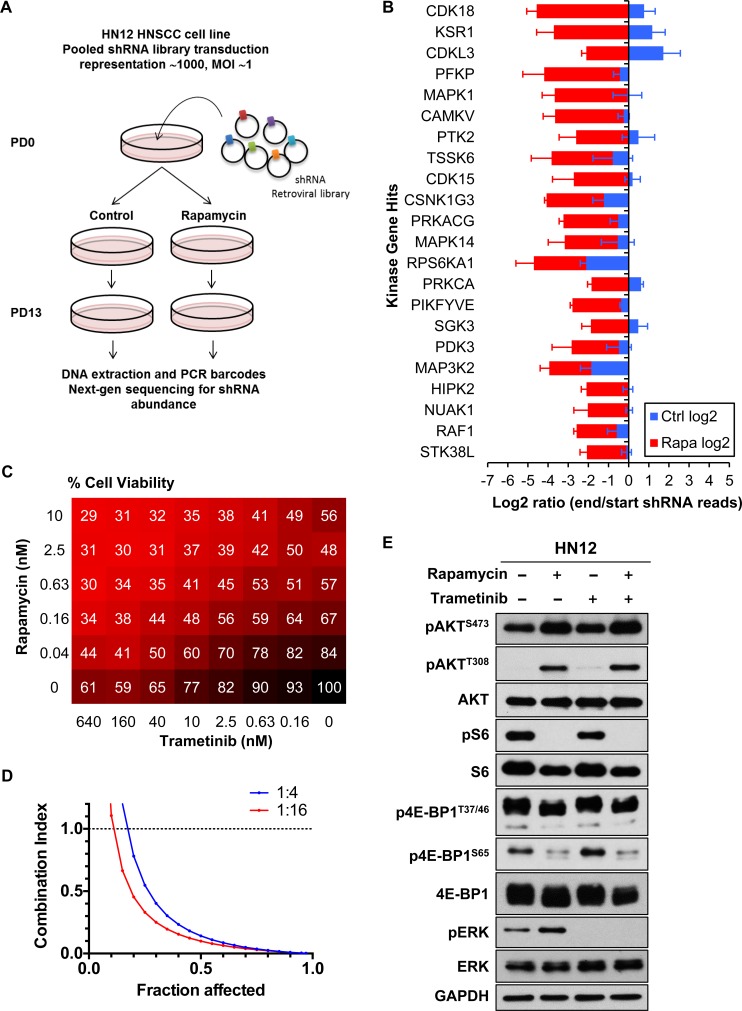
RNAi synthetic lethal screen **A.** Schema of shRNA screen. The change in the relative abundance of each shRNA in the library over time is measured using the normalized PD 13/PD 0 ratio of its reads. A log2 PD 13/PD 0 ratio of < 0 indicates the shRNA is depleted in the population over time, and a log2 PD 13/PD 0 ratio of < 0 indicates the shRNA is enriched in the population. **B.** Hit kinase genes. Targets were filtered by log2 PD 13/PD 0 ratio of < −2 (*n* = 3). **C.** Factorial dose matrix combinatorial drug treatment. HN12 cells were incubated for 72 hrs with indicated concentrations of drugs. Numbers on the matrix indicate % Cell Viability (*n* = 3). **D.** Computer-simulated Fa-CI curves were created based on the matrix data. The ratios of rapamycin : trametinib were indicated. Synergism (CI < 1), additive effect (CI = 1), or antagonism (CI > 1) for the indicated levels of growth inhibition (Fraction affected) induced by the drug combination. **E.** mTOR/ERK pathway. HN12 cells were treated with 0.1% DMSO, 20nM rapamycin, 20nM trametinib or the combination for 24hrs.

We utilized a pooled retroviral shRNA library for the synthetic lethal screen [21]. We analyzed the change in relative abundance of each shRNA over time by sequencing of half-hairpin (HH) barcodes derived from the shRNA population to identify those that are antiproliferative and are thus depleted from the population. We compared the lethality signature of the cells in presence of rapamycin and control to identify those shRNAs showing selective depletion in the cells in presence of rapamycin but not in control (Figure 1A). We chose the HNSCC cell line HN12 [16] for the screen, as this HNSCC cell line carries no driver mutations endogenously [9], but, as most HNSCC-derived cells, exhibits elevated mTOR activity and it is sensitive to growth inhibition in response to rapamycin [14]. Target genes were defined as hits when the difference between log2 end/start ratio of rapamycin treated samples and that of control samples was ≤ −2. We identified 117 genes as hits, among which 22 encoded kinases (Figure 1B). This gene set was particularly enriched for kinases involved in ERK pathway signaling, including KSR1 (*KSR1*), CRAF/RAF1 (*RAF1*), ERK (*MAPK1*) and RSK1 (*RPS6KA1*), which suggests that their depletion by shRNAs is synthetically lethal with rapamycin. We also performed gene ontology (GO) enrichment analysis and found the GO term “MAPK cascade” was significantly related to this gene set (*P* = 2.7E-8). These findings prompted us to explore the impact of ERK signaling inhibition in HNSCC cells in combination with rapamycin. As an approach, we took advantage that a selective MEK1/2 inhibitor trametinib, which blocks ERK activation, was recently approved for the treatment of unresectable or metastatic melanoma with *BRAF*^V600E^ or *BRAF*^V600K^ mutations by the US Food and Drug Administration as a monotherapy and in combination with dabrafenib [22].

In order to evaluate the effect of drug-drug interaction on cell viability, we performed a factorial dose matrix combinatorial drug treatment with rapamycin and trametinib. HN12 cells were incubated with these drugs in a 6 × 8 dose-response matrix and cell viability was measured after 72 hrs of treatment (Figure 1C). We also investigated whether the combination effect displayed a synergistic activity. For this purpose, the fraction affected (Fa) combination index (CI) plot (Fa-CI plot) curves were simulated using CompuSyn software [23]. CI at 0.5 of Fa was 0.14 and 0.10 for 1:4 and 1:16 constant ratio of rapamycin to trametinib respectively (Figure 1D). These data indicated a strong synergism of the combination therapy in HN12 cells. We next evaluated the effect of rapamycin and trametinib on the ERK and PI3K-mTOR signaling pathway in these HNSCC cells. For this purpose, cells were seeded in 6-well plates and harvested after 24h treatment, and samples were used for western blotting analysis. As expected, rapamycin decreased phospho-S6 (pS6) and phospho-4E-BP1 (p4E-BP1) (Figure 1E). In contrast, phospho-AKT (pAKT) and phospho-ERK (pERK) were increased (Figure 1E). Instead, trametinib clearly decreased pERK, consistent with its MEK blocking activity, and of interest, trametinib also partially decreased pS6 (Figure 1E).

### Synergistic effects of the combination of rapamycin and trametinib on HNSCC harboring *HRAS* and *PIK3CA* mutations

**Figure 2:**
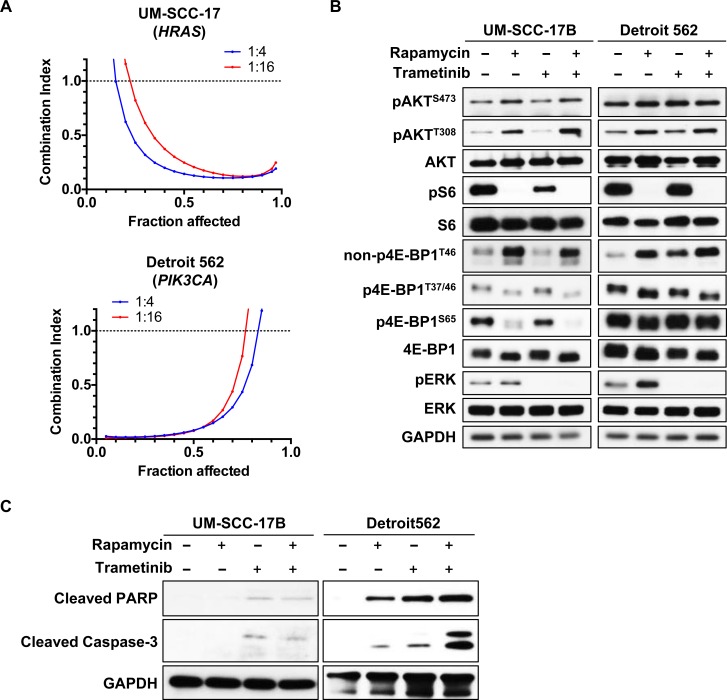
Synergistic effects of the combination of rapamycin and trametinib on HNSCC-derived cells harboring HRAS or PIK3CA mutations **A.** Computer-simulated Fa-CI curves were created based on the factorial dose matrix combinatorial drug treatment. The ratios of rapamycin : trametinib were indicated. Synergism (CI < 1), additive effect (CI = 1), or antagonism (CI > 1) for the indicated levels of growth inhibition (Fraction affected) induced by the drug combination. **B.** mTOR/ERK signaling pathway. Cells were treated with 0.1% DMSO, 20nM rapamycin, 20nM trametinib or the combination for 24hrs. **C.** Immunoblot analysis for cleaved-PARP and cleaved caspase-3. Cells were treated with 0.1% DMSO, 20nM rapamycin, 20nM trametinib or the combination for 24hrs.

HNSCC lesions often harbor activating mutations in *PIK3CA* (18.1 %), encoding the catalytic PI3K-α subunit and less frequent oncogenic mutants of the *HRAS* (5.6 %) or *KRAS* (0.2 %) genes [8, 12], collectively referred herein as *RAS*. To explore whether the combination of rapamycin and trametinib also displays synergistic effect in HNSCC tumors harboring activating mutations of *RAS* and *PIK3CA*, we evaluated the impact of this drug combination in HNSCC cells harboring endogenous oncogenic mutations. We used UM-SCC-17B, which has a *HRAS* Q61L mutant and Detroit 562 cells exhibiting the *PIK3CA* H1047R mutation. The combination of rapamycin and trametinib demonstrated strong synergism in growth inhibition of these cells (Figure 2A, Supplementary Figure S1A). We next evaluated the effect of the combination treatment in the downstream signaling pathway of RAS and PI3K in these cell lines. Rapamycin decreased pS6 and p4E-BP1, but in contrast, pAKT and pERK were both increased (Figure 2B). Trametinib treatment clearly decreased pERK, and it also enhanced the rapamycin-mediated decrease of pS6 and p4E-BP1 (Figure 2B). We also evaluated whether trametinib, rapamycin, or their combination can promote apoptosis as judged by the accumulation of cleaved caspase-3. Remarkably, we found that whereas these drugs alone have limited effect, the combination treatment induced a significant increase in apoptosis in *PIK3CA* mutant HNSCC cell line, Detroit 562, but not in the *HRAS* mutant HNSCC cells UM-SCC-17B (Figure 2C).

### Antitumoral activity of the combination therapy with rapamycin and trametinib against HNSCC harboring *HRAS* or *PIK3CA* mutations

**Figure 3:**
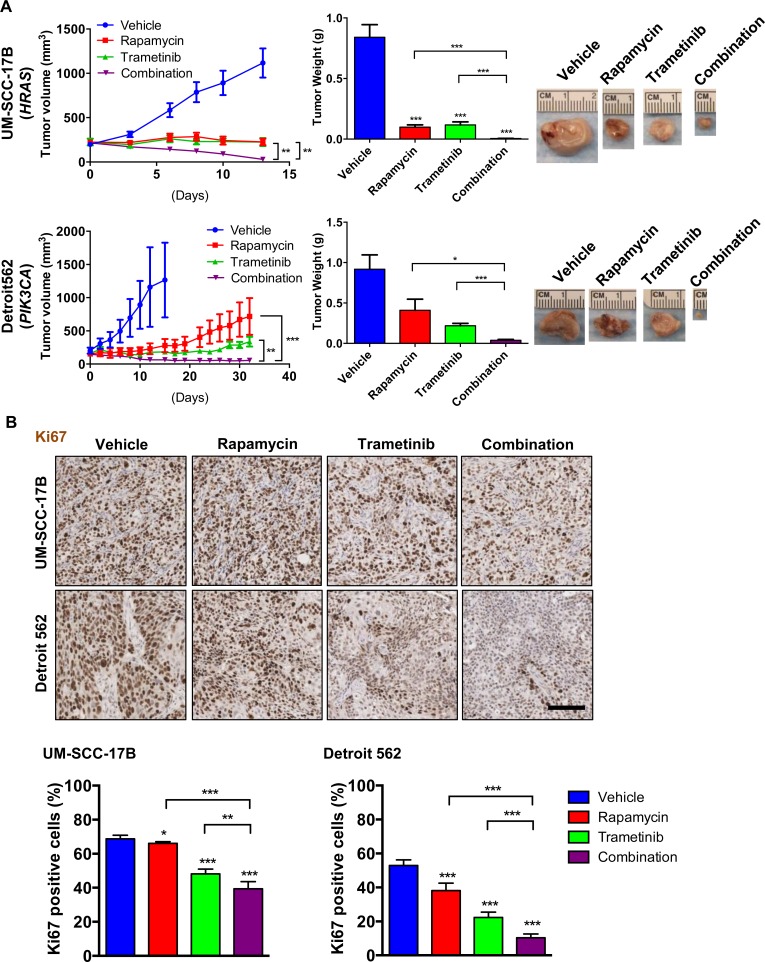
Antitumoral activity of the mTOR ERKcombination therapy with rapamycin and trametinib against HNSCC harboring HRAS or PIK3CA mutations **A.** Antitumor efficacy of rapamycin, trametinib, and combination. Athymic nude mice were transplanted with HNSCC cells. Treatment was initiated when the tumor volume reached approximately 200 mm^3^. The tumor growth curves (left), tumor weights at the end of the single agent and combined treatments (middle) and pictures of representative tumors (right) are displayed. Data points represent mean values ± SE (*n* = 10 per each group). **B.** Representative tumor tissue sections (top) and quantification (bottom) stained for Ki67 (*n* = 6 per each group). Scale bars represent 100 μm. *P < 0.05, **P < 0.01, ***P < 0.001.

**Figure 4:**
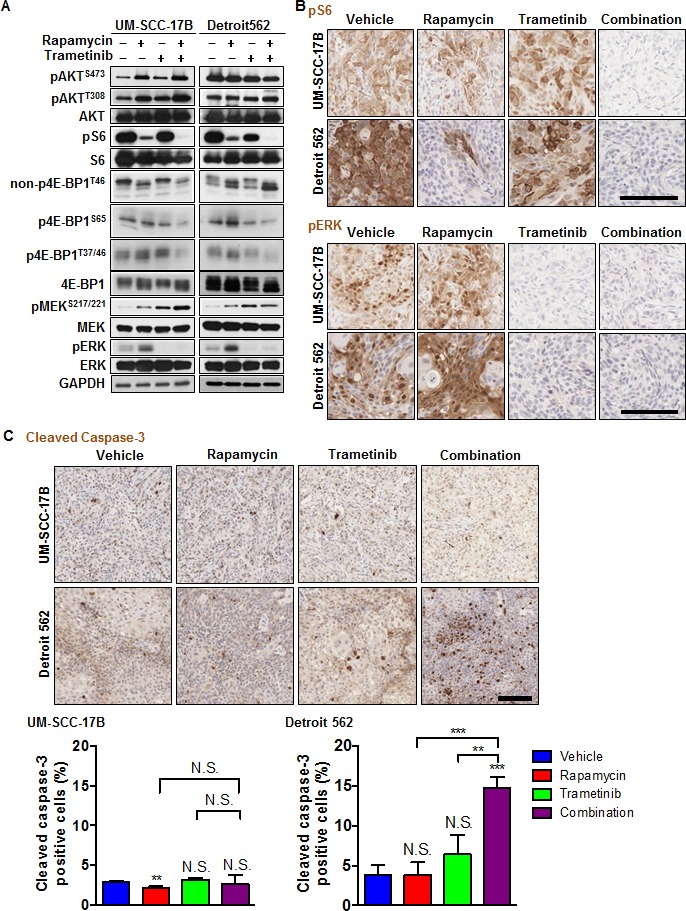
Effects of the combination of rapamycin and trametinib on mTOR/ERK signaling and apoptosis in HNSCC harboring HRAS or PIK3CA mutations **A.** mTOR/ERK signaling pathway. Cells were transplanted into athymic mice and treated with vehicle, rapamycin, trametinib or combination for four days. Tumor lysates were analyzed with immunoblot analysis. **B.** Representative tumor tissue sections stained for pS6 and pERK. Scale bars represent 100 μm. **C.** Representative tumor tissue sections (top) and quantification (bottom) stained for cleaved-caspase 3 (*n* = 3 per each group). Scale bars represent 100 μm. *P < 0.05, **P < 0.01, ***P < 0.001.

We next evaluated the antitumoral activity of the ERK-mTOR targeting combination therapy *in vivo*, using UM-SCC-17B and Detroit 562 HNSCC xenografts, which harbor endogenous activating *HRAS* and *PIK3CA* mutations, respectively. The combination therapy with rapamycin and trametinib was more effective than each of the single agents individually (Figure 3A). The body weight after 4 weeks treatment was 22.7±2.1 g in the combination treatment group and 23.7±0.7 g in vehicle control group. The antitumoral activity was reflected by a decreased immunohistochemical detection of nuclear Ki67, which was used for the evaluation of cell proliferation. The MEK-mTOR combination therapy showed significant reduction of HNSCC cells expressing nuclear Ki67 in both xenograft models (Figure 3B). Immunohistochemistry analysis of treated tumors revealed that trametinib enhanced the rapamycin-mediated inhibition of S6 and 4E-BP1 *in vivo*, and blunted the rapamycin-induced ERK activation in these HNSCC cells (Figure 4A, 4B). Consistent with the activation of ERK, rapamycin also induced elevated phospho-MEK1/2 (pMEK1/2) levels. pMEK1/2 was increased by trametinib, likely as a feedback mechanisms stimulating the activation of MEK upstream kinases upon MEK inhibition by its direct blocker. AKT was activated by rapamycin in UM-SCC-17B but not in Detroit 562 *in vivo*, suggesting that activation of the ERK pathway may represent a more general secondary effect of rapamycin than pAKT increase (Figure 4A). In addition, a clear distinction between these HNSCC cells was that cleaved caspase-3 was significantly increased by the combination therapy in Detroit 562 (*PIK3CA* mutant) but not in UM-SCC-17B (*HRAS* mutant) (Figure 4C).

### mTOR and MEK inhibition display a synergistic growth inhibitory activity in HNSCC cells genetically engineered to express activating *KRAS* and *PIK3CA* mutations

**Figure 5:**
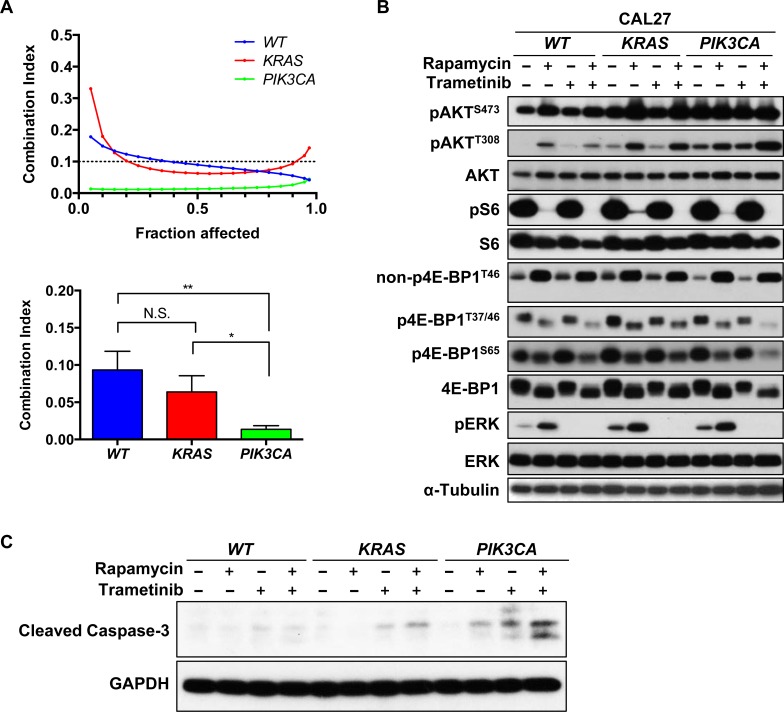
Synergism of rapamycin and trametinib in genetically engineered HNSCC cells to express activating KRAS or PIK3CA mutations **A.** Computer-simulated Fa-CI curves were created based on the factorial dose matrix combinatorial drug treatment. The ratios of rapamycin : trametinib were 1:16. Synergism (CI < 1), additive effect (CI = 1), or antagonism (CI > 1) for the indicated levels of growth inhibition (Fraction affected) induced by the drug combination (left). CI values at Fa = 0.5 was used to calculate the mean between experiments (*n* = 3). *P < 0.05, **P < 0.01. **B.** mTOR/ERK signaling pathway. Cells were treated with 0.1% DMSO, 20nM rapamycin, 20nM trametinib or the combination for 0.5h or 24hrs. **C.** Immunoblot analysis for cleaved-caspase 3. Cells were treated with 0.1% DMSO, 20nM rapamycin, 20nM trametinib or the combination for 24hrs.

Our observations suggested that mTOR and MEK co-targeting exerts a synergistic antiproliferative effect in HNSCC cells *in vitro* and *in vivo*, and a selective pro-apoptotic impact on *PIK3CA* expressing HNSCC cells. However, cancer cells display an intrinsic heterogeneity, hence it is unclear if trametinib and rapamycin promote apoptosis in Detroit 562 because these cells express mutant *PIK3CA* or if this response is due to cell-dependent variations. As an approach to explore these possibilities, we next investigated whether a significant synergism was displayed in isogenic HNSCC cells, CAL27 cells, which were genetically engineered to express *RAS* and *PIK3CA* mutants [17]. Strong synergism was demonstrated between trametinib and rapamycin in wild type CAL27 cells and in both *KRAS* and *PIK3CA* expressing CAL27 cells, supporting that the drug combination is effective in HNSCC cells displaying multiple genetic alterations (Figure 5A, Supplementary Figure S1B). In this biological relevant system, *PI3KCA* expressing CAL27 cells showed significantly stronger synergism than CAL27 *WT* and CAL27 *KRAS* cells. Indeed, CI at ED_50_ was 0.09, 0.06 and 0.01 for CAL27 *WT*, CAL27 *KRAS* and CAL27 *PIK3CA* cells, respectively (Figure 5A).

We next evaluated the effect of rapamycin and trametinib in the downstream signaling pathway elicited by RAS and PI3K in these cells. As for other HNSCC cells, rapamycin decreased pS6 and p4E-BP1 in these cell lines. In contrast, pAKT and pERK were increased (Figure 5B). Trametinib clearly decreased pERK and enhanced the rapamycin-mediated reduction of pS6 and p4E-BP1 (Figure 5B). We also evaluated apoptosis by cleaved caspase-3 (Figure 5C). Rapamycin increased caspase-3 cleavage in CAL27 *PIK3CA* cells but failed to stimulate apoptosis in the CAL27 *WT* cell line. Trametinib also promoted a limited increase in cleaved caspase-3 in CAL27 *KRAS*, and this effect was much more remarkable in CAL27 *PIK3CA* cells (Figure 5C).

### Antitumoral activity of the rapamycin and trametinib combination therapy in genetically engineered HNSCC cells expressing activating *RAS* or *PIK3CA* mutations

**Figure 6:**
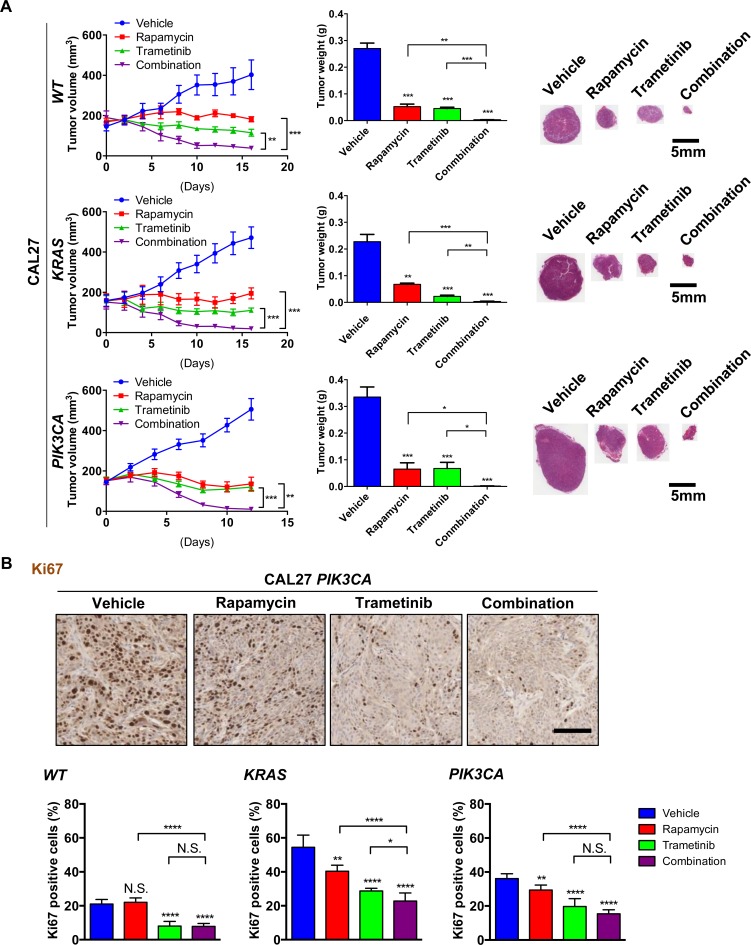
Antitumoral activity of the rapamycin and trametinib combination therapy against genetically engineered HNSCC cells expressing KRAS and PIK3CA oncogenes **A.** Antitumor efficacy of rapamycin, trametinib, and combination. Athymic nude mice were transplanted with HNSCC cells. Treatment was initiated when the tumor volume reached approximately 200 mm^3^. The tumor growth curves (left), tumor weights at the end of the single agent and combined treatments (middle) and representative histological sections for each treatment group. Scale bars represent 5 mm (right) are displayed. Data points represent mean values ± SE (*n* = 10 per each group). **B.** Representative tumor tissue sections (top) and quantification (bottom) stained for Ki67 (*n* = 6 per each group). Scale bars represent 100 μm. *P < 0.05, **P < 0.01, ***P < 0.001.

**Figure 7:**
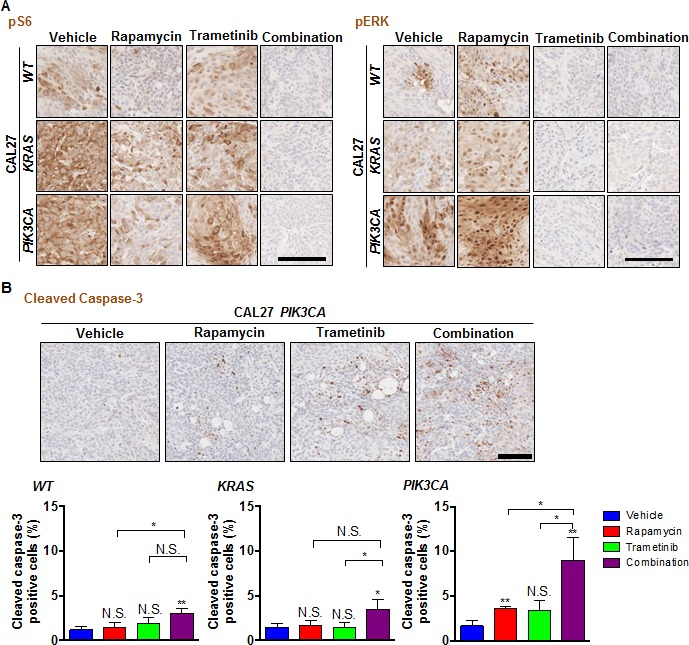
Effects of the combination of rapamycin and trametinib on mTOR/ERK signaling and apoptosis in genetically engineered HNSCC cells KRAS and PIK3CA mutations **A.** Representative tumor tissue sections stained for pS6 and pERK. Scale bars represent 100 μm. **B.** Representative tumor tissue sections (top) and quantification (bottom) stained for cleaved-caspase 3 (*n* = 3 per each group). Scale bars represent 100 μm. *P < 0.05, **P < 0.01.

We next investigated whether the combination of rapamycin and trametinib was effective in the isogenic cell line panel CAL27 *WT*, CAL27 *HRAS* and CAL27 *PIK3CA* xenograft models. A more significant antitumoral effect was observed in the groups treated with the combination therapy than in the groups treated with single agents in each of these xenograft models (Figure 6A). This was reflected by decreased nuclear Ki67, a biomarker for cell proliferation, in the combination therapy compared to single agents (Figure 6B, Supplementary Figure S2A). To study the biochemical and biological effects of the drug combination *in vivo* in this isogenic HNSCC cell panel, we performed immunohistochemistry for pS6, pERK (Figure 7A) and cleaved caspase-3 (Figure 7B). Rapamycin mediated inhibition of pS6 was enhanced by the combination with trametinib (Figure 7A). ERK was activated by rapamycin in these cells, and strongly inhibited by the therapy with trametinib (Figure 7A). Cleaved caspase-3 was significantly induced in the tumors treated with the combination therapy in CAL27 *PIK3CA* (Figure 7B). However, only minor pro-apoptotic effects were observed in CAL27 *WT* and CAL27 *KRAS* (Figure 7B, Supplementary Figure S2B). These finding suggest that while co-targeting MEK and mTOR is effective for tumor reduction in all HNSCC models tested, *PIK3CA* expression specifically sensitizes HNSCC cells to the pro-apoptotic activity of MEK and mTOR combined targeted treatment, thereby displaying increased response.

## DISCUSSION

Our *in vitro* data demonstrated strong synergism of the combination treatment with rapamycin and trametinib against HNSCC -derived cells exhibiting quite distinct genomic alterations, including cells carrying endogenous and genetically engineered *RAS* or *PIK3CA* mutations. Furthermore, the co-targeting therapy demonstrated significant effect on tumor growth in all HNSCC xenograft models tested. Intriguingly, CAL27 engineered to express *PIK3CA* mutations displayed the strongest synergism *in vitro*. Furthermore, the combination treatment significantly induced cleaved-caspase 3 in HNSCC cell lines carrying *PIK3CA* mutation endogenously and when ectopically expressed. Thus, in the presence of *PIK3CA* mutations, adding MEK inhibitors to mTOR blockade causes a proapoptotic switch from cytostatic to cytotoxic, which has important beneficial therapeutic implications.

The mechanism of this remarkable genotype-specific drug interaction remains unclear. For example, the ERK pathway has profound effects on the regulation of cell survival pathways by the post-translational phosphorylation of pro-apoptotic and apoptotic regulatory molecules, including BAD, BIM, Mcl-1, caspase 9 and Bcl-2 [24]. On the other hand, mTOR also possesses both pro-apoptotic and anti-apoptotic effects [25]. Activated S6K is capable of binding to mitochondrial membrane and phosphorylating the pro-apoptotic molecule BAD, rendering BAD inactive and promoting cell survival [26]. In line with these observations, mTOR inhibition can promote apoptosis in several cancer models [16, 27]. Conversely, several studies demonstrated that mTOR inhibition attenuates cytotoxic agent-induced apoptosis in malignant cells [28, 29]. The mTOR function on apoptosis appears to be dictated by cell type and its activation state as well as by the mutational status of its downstream targets including well known apoptosis-regulatory proteins such p53, BAD and Bcl-2 [30]. Thus, the interplay between mTOR and ERK inhibition may have pro-apoptotic functions in restricted genetic contexts. In line with this possibility, recently, Hata *et al.* reported that the concomitant use of MEK and PI3K inhibitor lead to upregulation of PUMA and BIM, both of which were necessary for the induction of an apoptotic response in *KRAS*-mutant in another cancer type, non-small cell lung cancer (NSCLC) [31, 32]. Certainly, the underlying reasons in HNSCC that an apoptotic response is only elicited in *PIK3CA* mutant cells is clearly clinically relevant, and its precise underlying mechanism warrants further investigation.

In the current study we identified 22 kinases whose suppression is synthetic lethal with rapamycin for therapy against HNSCC. These include KSR1 (*KSR1*), CRAF/RAF1 (*RAF1*), ERK (*MAPK1*) and RSK1 (*RPS6KA1*), which are involved in the ERK signaling pathway, suggesting that interfering with ERK activation renders HNSCC cells more sensitive to mTOR inhibition. Indeed, we show here that co-targeting MEK and mTOR using FDA approved therapeutic agents, trametinib and rapamycin, respectively, exerts a synergistic anti-proliferative and pro-apoptotic effect in all HNSCC cell lines tested irrespective of their quite distinct mutational genomic landscape [9]. Furthermore, we found that the combined drug therapy was much more effective in promoting tumor regression that any of the single agents alone, supporting that synthetic gene lethality screens can be exploited for the development of new therapeutic options for HNSCC treatment.

In addition to the ERK pathway, we also identified p38 MAPK (*MAPK14*) and MAP3K2 (*MAP3K2*), which are involved in other MAPK pathways as co-targeting candidates. Recently, we reported that p38 MAPK functions as a positive regulator of HNSCC in the context of the tumor microenvironment, controlling cancer cell growth and tumor-induced angiogenesis and lymphangiogenesis [33]. We also identified kinases involved in PI3K/AKT pathway. Among these were SGK3 (*SGK3*), ARK5/NUAK1 (*NUAK1*), and NDR2 (*STK38L*). For example, activation of SGK3 downstream of PIK3CA and INPP4B is required for 3D cell proliferation, invasive migration, and tumorigenesis *in vivo* [34]. Overexpression of ARK5 confers tolerance to glucose starvation, which is a stress that leads to a decrease in ATP and an increase in AMP [35]. Activated AKT stimulates cell invasion by phosphorylating ARK5 at Ser600 [35]. ARK5 has been also reported to act as a tumor invasion-associated factor through NDR2 during IGF-1 signaling [36]. We also identified FAK (*PTK2*) and HIPK2 (*HIPK2*) in our synthetic lethality screens. FAK promotes cell survival through kinase-dependent and kinase-independent mechanisms [37, 38]. Overall, these results suggest that several MAPK and PI3K/AKT pathway components and FAK, among others, may participate in adaptive response mechanisms promoting resistance to rapamycin in HNSCC cells. Hence, their knock down or pharmacological inhibition with small molecule inhibitors can be also considered for the future development of more effective combination therapies for HNSCC.

Overall, our unbiased genetic screen revealed that co-targeting mTOR and ERK using FDA approved agents results in a remarkable synergistic interaction in HNSCC preclinical models. While the inhibition of the PI3K/AKT/mTOR and ERK pathways has been investigated in other malignancies, the *in vivo* impact of this co-targeting strategy for HNSCC has not been well validated. Indeed, in all HNSCC tumors tested, the combination of tramatinib and rapamycin caused a rapid tumor collapse, and was much more active than any of these agents alone. Furthermore, we now show that the concomitant inhibition of mTOR and MEK promotes the apoptotic demise of HNSCC cells specifically in tumors expressing the *PIK3CA* oncogene, the most frequently driver mutation in this cancer type [8, 12]. Thus, these findings will have significant clinical implications, as they define a new mechanism-based precision therapeutic approach for the treatment of HNSCC patients, and define *PIK3CA* as a suitable biomarker of heightened beneficial therapeutic response to the combined treatment.

## MATERIALS AND METHODS

### Cell lines, tissue culture and antibodies

Human head and neck cancer cell lines Cal27, Detroit 562, were from American Type Culture Collection; UM-SCC-17B cell line was from Thomas Carey, University of Michigan. Cells were cultured in Dulbecco's modified Eagle's medium with 10 % fetal bovine serum supplemented with antibiotics, 5 % CO_2_ at 37°C. All cell lines underwent DNA authentication by multiplex STR profiling (Genetica DNA Laboratories, Inc. Burlington, NC) prior to the described experiments to ensure consistency in cell identity. Antibodies against AKT, pAKT^S473^, pAKT^T308^, ERK1/2, pERK1/2, S6, pS6, MEK1/2, pMEK1/2, 4E-BP1, non-p4E-BP1^T46^, p4E-BP1^T37/46^, p4E-BP1^S65^, cleaved caspase-3, cleaved PARP, α-Tubulin-HRP, GAPDH were purchased from Cell Signaling Technology (Beverly, MA). Mouse anti-Ki67 antibody was purchased from DAKO (Carpinteria, CA).

### shRNA screening

shRNA screening was performed as described [21]. HN12 cells were infected with pools of retroviral shRNA at a representation of ∼1,000 and a multiplicity of infection (MOI) of ∼1. At day 3 post infection cells were selected with puromycin for an additional 3 days (1 μg/ml) to remove the minority of uninfected cells. After that, cells where propagated in culture for 3 days and then an initial population-doubling 0 (PD 0) sample was taken. The rest of the population was divided in 6 groups, and 3 groups where kept as a control and 3 where treated with rapamycin (100 nM). Cells where propagated in the presence or not of drug for an additional 12 doublings before the final, PD 13 sample was taken. For each passage a minimal representation of 1000 was maintained. The shRNA library contained 9,149 retroviral shRNAs targeting human kinases, phosphatases, genes involved in protein ubiquination, and genes implicated in cancer. The libraries where expressed using the retroviral vector MSCV-PM. For PD 0 and PD 13 samples, shRNA HH barcode was PCR-recovered from genomic samples and samples where sequenced to calculate abundance of the different shRNA probes.

The change in the relative abundance of each shRNA in the library over time is measured using the normalized PD 13/PD 0 ratio of its reads. A log2 PD 13/PD 0 ratio of < 0 indicates the shRNA is depleted in the population over time, and a log2 PD 13/PD 0 ratio of < 0 indicates the shRNA is enriched in the population. To identify shRNAs that are synthetically lethal with rapamycin, the mean log2 PD 13/PD 0 ratios of the rapamycin treated cell triplicates were compared to that of the control triplicates to derive the log2 ratio difference. A p-value of the difference between the two triplicates was calculated using the *t*-test. Targets were filtered by the presence of at least 2 different shRNAs for the same gene and a *P* < 0.05. GO enrichment analysis was performed with ToppGene Suite software[39].

### Cell growth assays and drug combination analysis

Alamar Blue Cell Viability Reagent was purchased from Life Technologies. (Grand Island, NY) Cells were cultured in 96-well-plate and treated with drugs for 72 hours. The manufacturer's instructions were followed to complete the assay. Drug synergy was determined by the combination index methods using CompuSyn software [23]. CI values less than 1, equal to 1, and greater than 1 represent synergism, additivity, and antagonism, respectively.

### Immunoblot analysis

The cells and the tissues were lysed in RIPA buffer containing Halt protease and phosphatase inhibitor cocktail (Thermo Scientific, Rockford, IL). Protein concentration was measured by Bio-Rad Protein Assay (Bio-Rad, Hercules, CA). Equal amounts of total proteins were subjected to SDS-polyacrylamide gel electrophoresis and transferred to PVDF membranes. Membranes were blocked with 5% nonfat dry milk in T-TBS buffer (50 mM Tris/HCl, pH 7.5, 0.15 M NaCl, 0.1% [v/v] Tween-20) for 2 h, and then incubated with primary antibodies in blocking buffer at 4°C overnight. Detection was conducted by incubating the membranes with horseradish peroxidase-conjugated goat anti-rabbit IgG secondary antibody (Southern Biotech, Birmingham, AL) was used at a dilution of 1:50,000 in 5 % milk-T-TBS buffer, at room temperature for 1 h, and visualized with Immobilon Western Chemiluminescent HRP Substrate (Millipore, Billerica, MA).

### Tissue analysis

All samples were fixed in zinc formalin (Z-Fix, Anatech) and embedded in paraffin; 5 μm sections were stained with Hematoxylin-Eosin for diagnostic purposes. For immunohistochemistry (IHC) studies the sections were deparaffinized, hydrated through graded ethanols, and the endogenous peroxidase inhibited with 3 % H_2_O_2_ in 70 % ethanol. The slides were extensively washed with distilled water and antigen retrieval was performed by high temperature treatment with 10 mM citric acid in a microwave. After washing with water and PBS, the slides were successively incubated with the primary and secondary antibodies, and the ABC reagent (Vector Laboratories, Burlingame, CA). The reaction was developed with 3-3′-diamonobenzifdine under microscopic control. A Mouse on Mouse (M.O.M.) Basic Kit (Vector Laboratories, Burlingame, CA) was used in Ki67 staining to inhibit binding of secondary antibodies to mouse tissue.

### *In vivo* mouse experiments and analysis

All the mice studies were carried out according to National Institutes of Health (NIH) approved protocols (ASP #13-695) in compliance with the NIH Guide for the Care and Use of Laboratory Mice. To establish tumor xenografts, cells were transplanted into the flanks of athymic nude mice (female, four to six weeks old) (Harlan Laboratories, Inc., Indianapolis, IN), and when the tumor volume reached approximately 200 mm^3^, the mice were randomized into groups and treated by intraperitoneal injection (ip) with trametinib (1 mg/kg/day) and rapamycin (5 mg/kg/day), or control diluent (*n* = 10 per each group). Tumor volume was calculated by using the formula length × width × width/2. The mice were euthanized at the indicated time points and tumors isolated for histologic and immunohistochemical evaluation.

### Data and statistical analysis

Data were analyzed using GraphPad Prism version 6 (GraphPad Software, San Diego, CA). Comparisons between experimental groups were made using unpaired *t* test. *P* < 0.05 was considered to be statistically significant.

## SUPPLEMENTARY MATERIAL FIGURES


