# Supplementary material for: A synthetic-lethality RNAi screen reveals an ERK-mTOR co-targeting pro-apoptotic switch in PIK3CA+ oral cancers
Source: Oncotarget. 2016 Feb 13;7(10):10696–709. doi: 10.18632/oncotarget.7372 (PMC4905432; doi:10.18632/oncotarget.7372)
Supplement: Supplementary file 1 [file oncotarget-07-10696-s001.pdf]

A synthetic-lethality RNAi screen reveals an ERK-mTOR co-targeting pro-apoptotic switch in *PIK3CA*$^{+}$ oral cancers

Supplementary Material

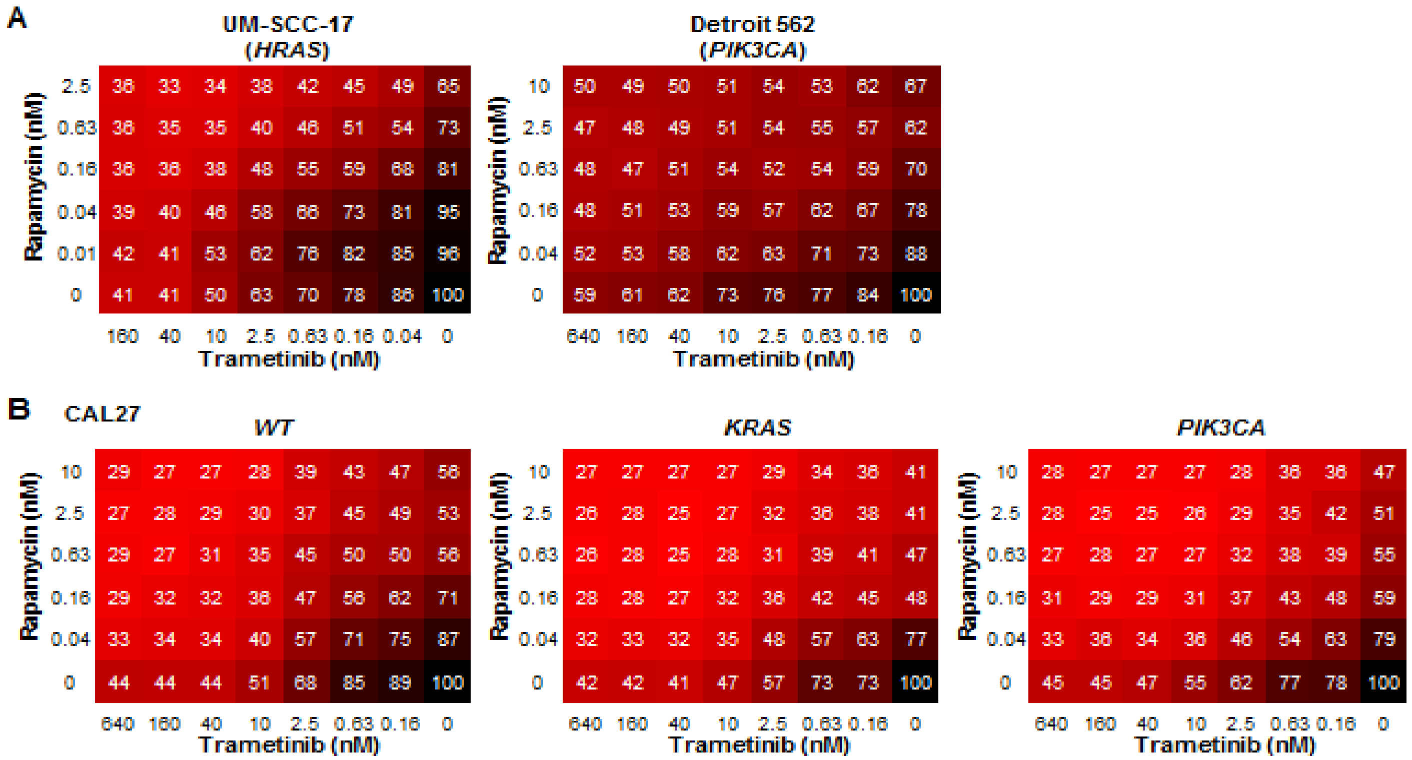

Supplementary Figure S1: Factorial dose matrix combinatorial drug treatment against UM-SCC-17B and Detroit 562 (A), and CAL27 *WT*, CAL27 *RAS* and CAL27 *PIK3CA* (B). Indicated cells were incubated for 72 hrs with indicated concentrations of drugs. Numbers on the matrix indicate % Cell Viability (n=3).

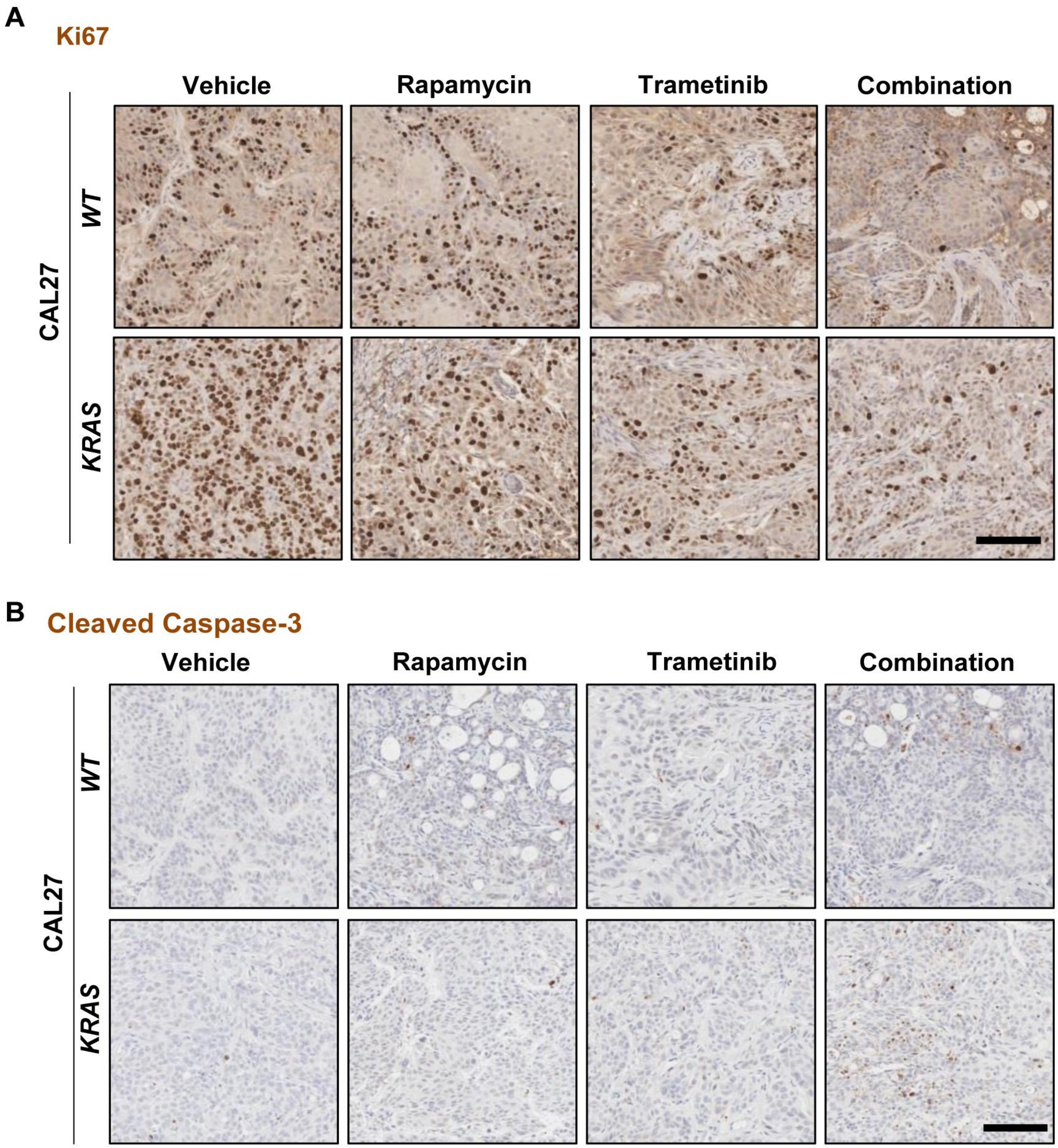

Supplementary Figure S2: Representative tumor tissue sections stained for Ki67 (A) and cleaved caspase-3 (B). Scale bars represent 100 μm.
